# Nature-Inspired Gold(I) Complexes as Anticancer Agents: Ligand Design, Structure–Activity Relationships, and Mechanisms

**DOI:** 10.3390/cancers18040631

**Published:** 2026-02-15

**Authors:** Amrin Begum, Navya PN, Pooran Kumar, Srinivasa Reddy Telukutla, Ruchika Ojha, Magdalena Plebanski, Suresh K. Bhargava

**Affiliations:** 1 Centre for Advanced Materials and Industrial Chemistry, School of Science, STEM College, RMIT University, Melbourne 3000, Australia; s3958644@student.rmit.edu.au (A.B.); s3628785@student.rmit.edu.au (N.P.); s3632495@student.rmit.edu.au (P.K.); ruchika.ojha@rmit.edu.au (R.O.); 2 Accelerator for Translational Research in Clinical Trials (ATRACT) Centre, School of Health and Biomedical Sciences, STEM College, RMIT University, Bundoora, Melbourne 3083, Australia; srinivasareddy.telukutla@rmit.edu.au

**Keywords:** natural products, gold(I) complexes, anticancer activity, heterobimetallic complexes

## Abstract

Nature-inspired ligands, including natural product scaffolds and bio-inspired motifs, have emerged as an important source for the development of gold(I) complexes with promising preclinical anticancer activity. This review focuses on structurally characterised gold(I) complexes derived from these ligands, emphasising how ligand structure and gold coordination influence the biological activity. This paper covers the synthesis of nature-inspired gold(I) complexes, alkynyl, N-heterocyclic carbene (NHC), heteroatom-donor, and heterobimetallic complexes, which collectively contribute to enhanced cytotoxicity, stability, and mechanistic diversity. By focusing on these nature-inspired gold(I) complexes, this review provides a concise structure–activity perspective and outlines future opportunities for rational design in anticancer research.

## Simple Summary

Gold complexes have emerged as promising anticancer agents due to their unique chemical properties and alternative mechanisms of action compared to traditional chemotherapy drugs. Their significance lies in their potential to overcome drug resistance and target cancer cells more selectively by targeting enzymes such as thioredoxin reductase, which is overexpressed in several cancer cells. In drug discovery, nature-inspired ligands provide structurally diverse and biologically relevant frameworks that enhance the activity and stability of gold complexes. Recent studies increasingly focus on gold(I) complexes derived from these ligands. In addition, heterobimetallic gold complexes have attracted attention for their synergistic therapeutic effects. Together, these systems represent an important direction in anticancer drug design.

## 1. Introduction

Cancer remains one of the leading causes of mortality worldwide, and its biological complexity continues to pose major challenges to modern medicine [1,2,3,4,5]. Current clinical strategies for cancer treatment include chemotherapy, radiation therapy, surgery, immunotherapy, and hormone therapy [6,7]. Among these, chemotherapy, based on the use of cytotoxic agents that target rapidly proliferating cells, has played a central role for decades [8]. Platinum-based drugs such as cisplatin have become widely used chemotherapeutic agents due to their broad-spectrum efficacy against multiple cancer types. However, despite their clinical success, platinum drugs are associated with severe side effects and the development of drug resistance, which significantly limits their long-term therapeutic effectiveness [9,10,11]. These drawbacks have intensified efforts to identify new chemotherapeutic agents with alternative mechanisms of action [12].

In this context, gold complexes continue to attract attention as potential anticancer therapeutics because they interact with biological targets distinct from those of platinum drugs [13,14,15]. Unlike cisplatin, which exerts its cytotoxicity primarily through covalent binding to DNA, gold(I) complexes target protein thiol- and selenol-containing enzymes, such as thioredoxin reductase (TrxR). This protein-directed mode of action is associated with distinct toxicity profiles and may offer improved selectivity by exploiting redox imbalances and enzyme overexpression characteristic of cancer cells [16,17,18]. Owing to the strong affinity of gold for sulphur and selenium, gold(I) complexes readily interact with cysteine and selenocysteine-containing enzymes, forming gold–thiolate or gold–selenolate species [19,20,21,22]. Among these, TrxR and glutathione reductase are of relevance, as they both play central roles in redox homeostasis and are frequently upregulated in malignant cells. Their inhibition disrupts cellular redox balance, promotes oxidative stress, and ultimately induces cancer cell death, highlighting the therapeutic potential of gold-based compounds as alternatives to conventional platinum chemotherapy [23,24,25,26,27]. Beyond classical TrxR inhibition, recent studies reveal that gold(I) complexes can induce diverse biological effects, including imaging-enabled functionalities [28,29].

Over the past two decades, gold(I) and gold(III) complexes have evolved from relatively simple coordination compounds into structurally sophisticated systems designed to incorporate biologically relevant motifs. Many of these developments are inspired by natural products, which have historically served as a rich source of clinically successful anticancer agents. Prominent examples include taxanes such as paclitaxel, vinca alkaloids such as vinblastine and vincristine, and camptothecin- and podophyllotoxin-derived drugs, all of which originate from plant or microbial secondary metabolites [30,31,32]. Natural product scaffolds offer remarkable structural diversity, intrinsic biological compatibility, and the potential for synergistic interactions when combined with metal centres.

Nitrogen-containing heterocycles, including quinazoline, pyrimidine, and quinoline frameworks, which are commonly found in nucleobases and natural alkaloids, are known for their ability to engage in hydrogen bonding and π–π interactions with nucleic acids and proteins [33,34]. Incorporation of such heterocycles into gold(I) complexes can enhance target engagement and modulate biological activity [35,36]. Similarly, phenylpropanoid-derived structures such as combrestatin contribute additional π-conjugated systems capable of engaging in aromatic stacking and hydrophobic interactions with biomolecular targets, enhancing the overall binding specificity [37]. Amino acid-based ligands further introduce biocompatibility and opportunities for selective biomolecular recognition, while N-heterocyclic carbene (NHC) ligands provide exceptional stability to gold(I) complexes in biological environments [38]. In addition to mononuclear systems, heterobimetallic gold(I) complexes have emerged as a promising strategy to diversify biological function further. By combining gold(I) with a second metal centre, such as ruthenium or iron, these architectures can exploit complementary modes of action, improved cellular uptake, or enhanced photophysical properties, thereby expanding the therapeutic and diagnostic potential of gold-based systems.

This review discusses the nature-inspired gold(I) complexes as anticancer agents at the preclinical stage with a focus on in vitro cytotoxicity, structure–activity relationships (SAR), and mechanistic insights for metallodrugs. In this review, the term “nature-inspired” refers to gold(I) complexes that incorporate natural product scaffolds and biologically inspired ligands that mimic structural and functional motifs found in natural systems. Adopting a design-driven perspective, representative chemical classes are used as case studies to illustrate how ligand architecture influences biological activity and mechanism of action in gold(I)-based anticancer agents. The literature survey for this review was conducted as a focused, topic-driven search through major scientific databases such as Web of Science, Scopus, PubMed, and ScienceDirect, using combinations of keywords including gold(I) complexes, bioactive gold compounds, NHC–gold complexes, amino acid-based gold complexes, heterobimetallic and anticancer activity. The selection of studies was guided by relevance to the design, structural features, and anticancer properties of bioactive gold(I) complexes, with emphasis on reporting biological evaluation against cancer models. This review is organised into five sections: (2) nitrogen-containing complexes (quinazoline, pyrimidine, and quinoline); (3) heterocyclic NHC-based ligands; (4) stilbene-derived frameworks exemplified by combretastatin analogues; (5) amino acid-based gold(I) complexes; and (6) heterobimetallic gold(I) complexes. By analysing these structural classes, this review aims to highlight how rational ligand design and coordination chemistry can be leveraged to modulate cytotoxicity, stability, and biological behaviour, thereby guiding the development of next-generation gold-based anticancer agents.

## 2. Nitrogen-Containing Gold(I) Complexes

### 2.1. Quinazoline-Derived Gold(I) Complex

Quinazolines are a class of nitrogen-containing heterocyclic alkaloids that are widely found in nature and are recognised as important scaffolds in medicinal chemistry [39,40,41]. Quinazoline derivatives can be adapted to structural modification and functionalisation, which has contributed to their broad range of reported biological activities [42,43,44,45], including anticancer, antibacterial, anti-inflammatory, antimalarial, and antihypertensive effects [42,43,46]. Owing to these properties, quinazoline-based frameworks have been extensively explored as pharmacophores in anticancer drug discovery.

**Figure 1:**
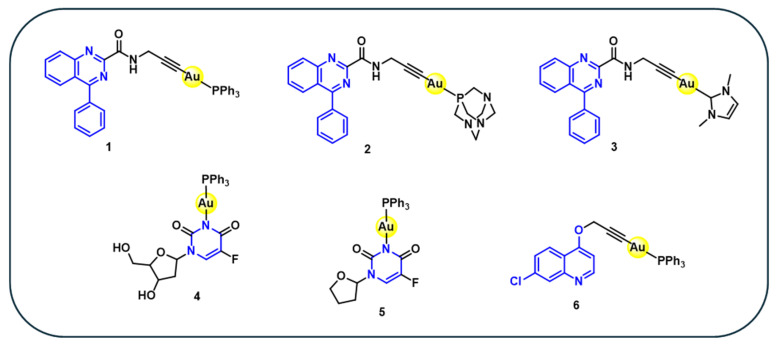
Structures of nitrogen-containing gold(I) complexes **1–6.**

In this context, three gold(I) complexes, **1–3** (Figure 1), bearing quinazoline carboxamide alkynyl ligands are synthesised by the reaction of the corresponding alkynyl quinazoline carboxamide with gold(I) precursors in the presence of a base [47]. The cytotoxic activity of these complexes is evaluated against human bladder cancer cell lines (5637 and T24). Complexes **1** and **2** exhibit significant antiproliferative activity against 5637 cells, with IC_50_ values of 0.17 µM and 2.59 µM, respectively (Table 1), whereas complex **3** showed only modest or negligible activity. Mechanistic investigations reveales that the cytotoxic effects of complexes **1** and **2** are associated with inhibition of TrxR, induction of reactive oxygen species (ROS), and activation of caspase-dependent apoptosis. Notably, although complex **3** also inhibit TrxR, it did not translate this enzymatic activity into effective cytotoxicity, highlighting that TrxR inhibition alone is insufficient to predict antiproliferative efficacy. Importantly, the corresponding free quinazoline carboxamide ligands are biologically inactive, confirming that the observed cytotoxicity arises from coordination to the gold(I) centre. Overall, these observations confirm the importance of ligand environment in modulating cytotoxic activity.

### 2.2. Pyrimidine-Conjugated Gold(I) Complexes

Pyrimidine is a naturally occurring nitrogen-containing heterocyclic compound, found to play an important role in biological systems and cancer pathogenesis [49,50,51]. The pyrimidine ring exhibits diverse pharmacological activity, where anticancer activity is mostly reported. Pyrimidines and their derivatives are functionally potent as the nitrogen atoms are readily bonded to DNA via hydrogen bonding, providing stability, thereby stabilising drug–DNA interactions in biological environments [52]. Substituted pyrimidines, such as 5-fluorouracil (5-FU) and its prodrug tegafur, have been potentially used to treat advanced cancers due to their ability to interfere with DNA and RNA synthesis [53]. In this context, two gold(I) complexes **4–5** (Figure 1) incorporating a pyrimidine ligand moiety are synthesised. The preliminary antitumor evaluation against murine leukaemia L1210-derived cells revealed that compound **4** exhibits activity comparable to tegafur, while compound **5** demonstrated enhanced activity. Complexes **4** and **5** react with methanethiol, *p*-toluenethiol, or imidazole, to form phosphine thiolate gold(I) complex or phosphine imidazolato gold(I)complexes, indicating that they possibly can react with a protein such as cysteine or histidine residue in vitro [54]. Although preliminary antitumor activity is reported in vivo, for these complexes, quantitative IC_50_ values are not provided in the study, limiting direct comparison with other gold complexes.

### 2.3. Quinoline Conjugated Gold(I) Complexes

Quinoline, a nitrogen-containing heterocyclic compound, is naturally found in many alkaloids such as cinchona alkaloids, echinopsine, vasicine, and numerous other alkaloids abundantly found in plants, animals, and microorganisms. Quinoline derivatives have long been considered as lead molecules in the field of drug design and development owing to their various biological activities, such as antimicrobial, antimalarial, and anticancer activities. Their heterocyclic core has been extensively modified to yield analogues with potent cytotoxicity [55,56,57]. Several quinoline-based compounds have already reached clinical application, while novel derivatives continue to attract attention for further development as anticancer agents. A quinoline conjugated gold(I) complex **6** (Figure 1) has been synthesised by coordination of propargyl ether quinoline ligand with gold(I) precursor in the presence of a base [48]. The cytotoxicity of complex **6** is moderate (IC_50_ = 1.5–10 μM) across the four cancer lines as compared with cisplatin (IC_50_ = 0.16–3.5 μM) (Table 1) [48]. These results indicate that while quinoline-based complexes show anticancer potential, further ligand optimisations are required to achieve anticancer activities comparable to clinically used drugs.

Collectively, these studies indicate alkynyl substitution influences cytotoxicity, with TrxR inhibition necessary but not sufficient for antiproliferative activity, highlighting the role of ligand-controlled cellular engagement.

## 3. Heterocyclic-Based Gold(I) Complexes

From a design perspective, heterocyclic ligands mark a shift from simple coordination motifs toward NHC–ligand frameworks, addressing key limitations such as ligand lability and insufficient stability under biological conditions. Therefore, this section highlights heterocycle-derived NHC systems as a case study illustrating how rational ligand design enhances gold(I) stability, cellular uptake, and anticancer potency. Heterocyclic compounds are widely distributed in nature and contain at least one heteroatom such as nitrogen, oxygen, or sulphur within a ring system. The presence of heteroatoms enables heterocycles to engage in hydrogen bonding, coordination interactions, and π-π stacking with biomolecular targets, contributing to high binding affinity and selectivity [36,58,59].

Among these, oxadiazoles are five-membered heterocyclic compounds containing one oxygen and two nitrogen atoms, which have received attention because of their broad spectrum of biological activities. The 1,2,4-oxadiazole heterocycle has been shown to exhibit diverse biological activities such as antibacterial, antiviral, anticonvulsant, antifungal, antidepressant, antiangiogenic, analgesic, anti-insomnia, anti-oedema, antiparasitic, and anti-Alzheimer effects [60]. Although relatively rare in nature, the 1,2,4-oxadiazole unit is present in some natural products, such as phidianidine A and phidianidine B, and quisqualic acid, which has inspired interest in its incorporation into bioactive gold complexes.

Recently, natural product-derived oxadiazole motifs have been integrated with imidazole-based NHC ligands to construct gold(I) complexes with enhanced biological potential. The imidazole ring serves as a versatile precursor for NHC formation, allowing for facile structural modification at the N-substituents. The rationale for replacing phosphine ligands with NHCs arises from the strong σ-donor properties of NHCs, which surpass those of traditional phosphines and significantly enhance the stability of gold(I) complexes [61,62,63,64]. Such stability is particularly advantageous in biological environments, where ligand dissociation can compromise therapeutic efficacy. Moreover, modification of the imidazole N-substituents provides an effective strategy to increase lipophilicity, tune steric demand, and enhance the donor strength of NHC ligands [63].

**Figure 2:**
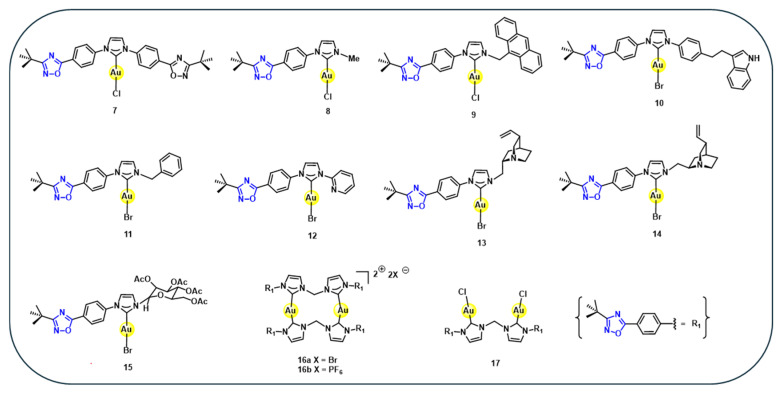
Structures of heterocyclic containing gold(I) complexes **7–17**.

Based on this design, a series of eleven gold(I) complexes **7–17** (Figure 2) are synthesised via in situ generation of the corresponding NHCs from imidazolium salts, followed by coordination to gold(I) precursors [63]. These gold complexes are evaluated in vitro against a panel of twelve human tumour cell lines and have shown strong anti-proliferative activity for several of these gold complexes. Notably, six complexes exhibit IC_50_ values below 0.1 µM and some individual IC_50_ values in the low nanomolar range, significantly outperforming cisplatin across different cancer cell lines (Table 2) [63]. These findings demonstrate that oxadiazole-containing NHC–gold(I) complexes represent a potent class of anticancer agents. Collectively, these results demonstrate that NHC-based heterocyclic ligands consistently outperform simpler donor systems, establishing ligand-controlled stability and electronic tuning as dominant drivers of anticancer activity in gold(I) complexes.

Notably, the corresponding free heterocyclic ligands generally exhibit weak or negligible cytotoxicity, whereas coordination to gold(I) enhances antiproliferative activity, often reducing IC_50_ values into the low nanomolar range. This enhancement arises from the synergistic combination of gold(I),–mediated redox activity, improved cellular uptake, and ligand-stabilised target engagement. The SAR emerges within the series, where increased lipophilicity and stronger σ-donating NHC ligands exhibit enhanced cytotoxicity. In contrast, complexes with bulkier or less electron-rich substituents exhibit reduced activity, indicating that fine electronic tuning of the carbene framework is critical for biological performance.

## 4. Stilbene-Derived Gold(I) Complexes

Stilbenes are phenylpropanoids characterised by a 1,2-diphenylethylene backbone, consisting of two aromatic rings connected by an ethylene bridge, and often bearing hydroxyl or methoxy substituents that confer metal coordination capabilities and biological activity [65,66]. Naturally occurring stilbene compounds such as resveratrol, combretastatin A-4, and pterostilbene are of significant interest in drug research and development due to their potential for both therapeutic and preventive applications. As a result, the medicinal chemistry of stilbene-based compounds has become a rapidly evolving field, encompassing a wide range of therapeutic areas [66,67]. Pharmacological studies have demonstrated that stilbenes exhibit a broad spectrum of biological activities, including anticancer, antimicrobial, antioxidant, anti-inflammatory, anti-diabetic, neuroprotective, anti-ageing, cardioprotective, and anti-neurodegenerative effects [68]. The presence of electron-donating groups, particularly hydroxyl moieties, facilitates chelation with transition metals, making them effective scaffolds for coordination chemistry. Their antioxidant and anticancer properties, along with their ability to chelate metals, make them attractive ligands for anticancer gold(I) complexes [69].

Moreover, stilbenes have shown extraordinary potential in the prevention and treatment of multiple diseases, including cancer, owing to their low in vivo toxicity combined with their ability to activate cell death pathways, and modulate oxidative and inflammatory responses [70]. The role of stilbene-conjugated gold(I) complexes ligands in anticancer drug development is discussed below.

### Combretastatin-Derived Gold(I) Complexes

**Figure 3:**
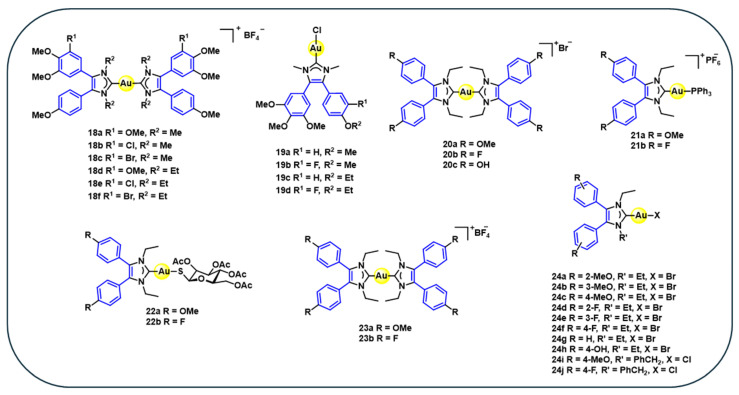
Structures of stilbene-derived gold(I) complexes **18–24**.

Combretastatin (combretastatin A-4) is a naturally occurring cis-stilbene specifically known for its antitubulin and anti-vascular properties, making it a potent antimitotic agent. Based on this established pharmacological profile, a series of gold(I) biscarbene complexes **18a–f** (Figure 3) are synthesised through the coordination of NHC ligands derived from combretastatin A-4 with gold(I) precursors via ligand exchange reactions [71]. Gold(I) biscarbene complexes exhibit strong antiproliferative activity across various cancer cell lines. Their cytotoxic potency depends on aromatic substitution patterns and N-alkylation of the NHC ligands. Methoxy-substituted complexes **18a** and **18d** show the highest activity against HT-29 colon carcinoma cells (IC_50_ = 0.06–0.08 µM) (Table 3). Chloro-substituted analogues **18b** and **18e** display enhanced efficacy in multidrug-resistant breast cancer MCF-7 cells (IC_50_ = 0.06 µM) (Table 3), while bromo-substituted complexes **18c** and **18f** exhibit broader but less selective cytotoxic profiles. All complexes maintain submicromolar activity against P-glycoprotein-overexpressing KB-V1 cells, indicating partial circumvention of drug-efflux-mediated resistance. The N-ethyl-substituted derivatives (**18d–f**) consistently outperform their N-methyl counterparts (**18a–c**), highlighting the influence of subtle ligand alkylation on biological performance [71].

**Figure 4:**
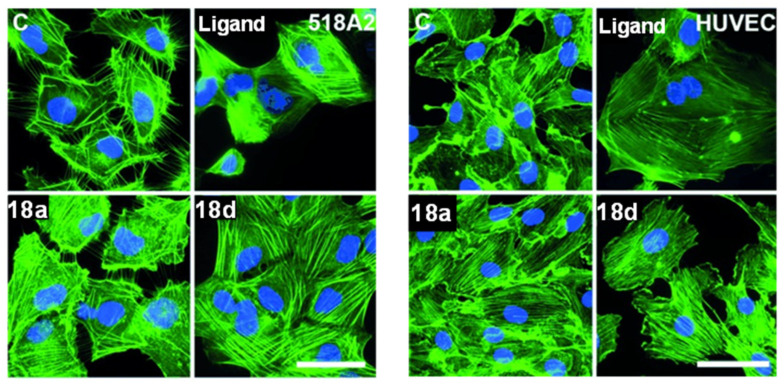
Fluorescence images of human melanoma (518A2) and human umbilical vein endothelial cells (HUVECs). 518A2 cells treated with control (C, 2.5 nM), ligand (250 nM), and complexes **18a** and **18d** (500 nM each). HUVECs incubated with ligand and **18a** (100 nM each), as well as **18d** (1µM) for 24 h. Fixed and stained with Alexa Fluor 488–phalloidin (1:100 dilution) F-actin is shown in green and nuclei in blue (DAPI). Excitation at 488 nm; emission collected at 500–550 nm. Scale bar: 50 µm [71]. (reproduced with permission).

The gold(I) biscarbene complexes derived from combretastatin A-4 **18a** and **18d** exhibit promising biomedical potential as multimodal anticancer agents. Unlike the parent compound CA-4, these gold complexes target the actin cytoskeleton instead of tubulin (Figure 4), inducing stress fibre formation and causing G1 cell cycle arrest. This shift in biological behaviour highlights an emerging trend in gold(I) coordination chemistry, where metal binding fundamentally reprograms cellular mechanisms beyond enhancing cytotoxic potency. They show potent antiproliferative activity against various cancer cell lines, including multidrug-resistant types, and suppress matrix metalloproteinases-2 (MMP-2) expression, which is key to metastasis. Additionally, they demonstrate strong antivascular effects both in vitro (tube formation inhibition in human umbilical vein endothelial cells, HUVECs) (Figure 4) and in vivo (CAM assay), while being well tolerated in mouse melanoma xenografts [71]. These findings demonstrate that coordination of bioactive natural product ligands to gold(I) can enhance ligand-centred fluorescence, while preserving their anticancer activity and offering additional opportunities for mechanistic studies.

While complexes **18a–f** directly incorporate the natural combretastatin scaffold, subsequent derivatives (**19–24**) (Figure 3) represent structurally expanded stilbene–gold(I) hybrids. These complexes introduce variations in the coordination sphere, such as phosphine, halide, and carbene ligands, to investigate how structural modifications influence stability and anticancer activity. Complexes **19a–d** are synthesised using N-methyl-4,5-diarylimidazolium salts to form silver–NHC intermediates, followed by transmetallation to yield the desired gold(I) complexes. Although these complexes demonstrate moderate cytotoxicity (IC_50_ = 6.9–50 μM) (Table 3), they are substantially more active than their metal-free analogues, which show negligible anticancer effects. Notably, these complexes did not inhibit tubulin polymerisation; instead, they showed distinct cellular uptake via copper as a control and organic cation transporters, suggesting a unique mechanism of action that differs from classical microtubule-targeting agents [72].

Building on the antitumour potential of N-methyl-4,5-diarylimidazolium-derived gold(I) carbene complexes, a new series of gold(I)–NHC complexes, **20–23** (Figure 3), featuring bis [1,3-diethyl-4,5-diarylimidazol-2-ylidene] ligands, is synthesised. Complexes **20**, **21,** and **23** are cationic, bearing 4-methoxy or 4-fluoro substituents on the aromatic rings. In analogy to the clinically relevant gold compound auranofin, the NHC ligands were further combined with triphenylphosphine **21a–b** and a sugar-derived thiolate ligand, 2′,3′,4′,6′-tetra-O-acetyl-β-D-glucopyranosyl-1-thiolate **22a–b**, to investigate the effects of mixed-ligand systems on biological activity. For synthesis, complexes **20–23** are synthesised using imidazolium salts as ligands, followed by silver-mediated carbene formation, transmetallation with gold(I) precursors, and subsequent alkylation or ligand exchange to yield the final products [73]. All these complexes exhibit potent cytotoxicity against cancer cell lines up to 10-fold lower than those of cisplatin and 5-FU. Notably, **20b** demonstrated an IC_50_ of 0.10 μM against MCF-7 cells, significantly outperforming cisplatin (IC_50_ = 1.6 μM) and 5-FU (IC_50_ = 4.7 μM) (Table 3). Unlike other gold(I) complexes, the mechanistic studies ruled out TrxR, estrogen receptor, and cyclooxygenase as primary targets, despite their relevance in the design rationale. Although the exact mode of action is unknown, evidence suggests involvement of mitochondrial pathways, cell cycle disruption, proteasome inhibition, and kinase modulation leading to apoptosis. Further investigations are necessary to explore the molecular targets and mechanisms underlying their anticancer activity [73].

Using the same ligand 4,5-diarylimidazoles, a series of neutral NHC–gold(I) halide complexes **24a–j** (Figure 3) are successfully synthesised via silver-mediated carbene generation from imidazolium salts, followed by transmetallation with gold(I) precursor [74]. The cytotoxicity results reveal that complexes **24a–j** exhibit high cytotoxicity across various cancer cell lines, with complex **24g** showing greater efficacy (IC_50_ = 0.87–3.1 μM) outperforming cisplatin (IC_50_ = 1.6–7.8 μM) (Table 3) [74]. Complexes with methoxy substitution **24a–c** have shown similar cytotoxicity, comparable to **24g**, while the complex with ortho-fluoro substitution **24d** has shown enhanced activity against MDA-MB-231. In contrast, complex **24h** with 4-OH substitution has resulted in low cytotoxicity. It is observed that variations in N-substituents have a minimal influence on activity [74]. Overall, the growth inhibitory effects were dependent on the presence of the gold centre, and most of these complexes outperformed cisplatin, highlighting the critical role of aryl substitution in modulating anticancer properties.

Compared to the parent stilbene derivatives, which typically exhibit modest antiproliferative effects, coordination to gold(I) enhances cytotoxic potency and is associated with mechanistic features such as cytoskeletal disruption and antivascular activity. These observations indicate that gold(I) acts as an active pharmacophore that reshapes the biological profile of the stilbene scaffold. Collectively, the stilbene–gold(I) complexes discussed reveal a coherent and multifactorial SAR governed by ligand substitution, gold coordination mode, and interaction with efflux transporters. Increased alkyl chain length at the nitrogen centre enhances cytotoxic potency, while specific aromatic substitutions modulate susceptibility to P-glycoprotein-mediated efflux, with halo substituents partially evading transporter recognition. Methoxy substitution generally supports cytotoxicity, whereas hydroxylation or certain electron-withdrawing groups can diminish activity depending on position. Importantly, coordination of stilbene ligands to NHC–gold(I) frameworks consistently improves both potency and uniformity of anticancer activity compared to ionic gold(I) analogues, highlighting ligand-controlled stability and transporter evasion as key determinants of efficacy within this class. Overall, the optimisation of stilbene–gold(I) complexes requires the integrated consideration of electronic effects, steric modulation, coordination mode, and efflux–transporter interactions.

Although TrxR inhibition and ROS generation are frequently reported for gold(I) complexes, NHC–stilbene-derived complexes additionally exhibit anticancer activity through alternative pathways, including mitochondrial dysfunction, cytoskeletal disruption, and anti-vascular effects. This mechanistic diversity highlights that gold(I) complexes should be viewed as multifunctional systems whose biological behaviour is strongly dictated by ligand architecture.

## 5. Amino Acid-Based Gold(I) Complexes

Amino acids, as naturally occurring ligands, possess amine, carboxylate, thiol, and imidazole functional groups, making them excellent chelating agents for metal ions. Their ability to coordinate through nitrogen, oxygen, or even side-chain donor atoms (such as sulphur in cysteine or imidazole in histidine) allows the formation of stable metal–amino acid complexes. These metal complexes often exhibit enhanced biological activity, including antimicrobial, antioxidant, anticancer, and enzyme-inhibitory effects. The importance of metal complexes derived from amino acids lies not only in their inorganic coordination chemistry, but also in their significant biological potential, as some amino acids themselves can exhibit antioxidant activity and are integral to the active sites of many proteins and enzymes, where they coordinate with metal centres to facilitate essential biochemical functions [75].

**Figure 5:**
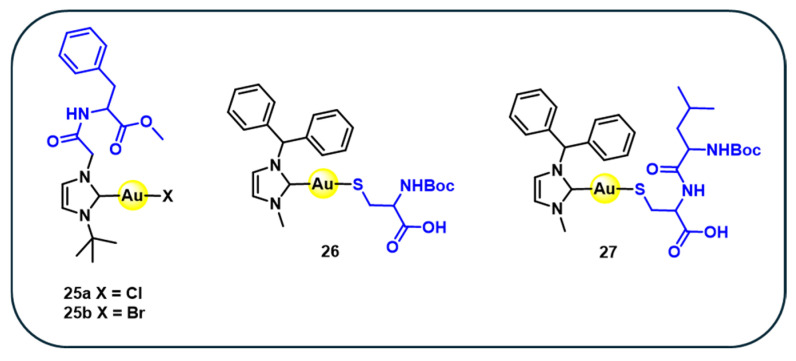
Structures of amino acid-based gold(I) complexes **25–27**.

A series of four gold–NHC complexes conjugated with amino acids **25–27** (Figure 5) is synthesised with the aurophilic behaviour of sulphur to couple a thiol-functionalised biomolecule to the gold centre [76]. The phenylalanine–NHC–gold(I) conjugate **25a** is prepared via transmetallation of an N-phenylalanine-substituted NHC–silver complex with gold(I) precursor [76]. Subsequent halide exchange affords the corresponding phenylalanine–NHC gold(I) bromide complex **25b**. Additionally, reaction of NHC gold chloride with *N*-Boc-protected cysteine methyl ester (Boc-Cys-OMe) or the dipeptide *N*-Boc-Leu-Cys-OMe leads to the formation of sulphur-coordinated (NHC)Au-S complexes, yielding the amino acid and phenyl aniline dipeptide derivatives **27** and **28**, respectively [76]. The cytotoxicity data shows that complexes **25a** display only moderate activity across the tested cell lines, with IC_50_ values that remain several times higher than those of cisplatin. In contrast, complex **26** stands out as the most active among this subgroup, achieving IC_50_ values in the low micromolar range and approaching the potency of cisplatin in HeLa and HT-29, although it remains less effective than cisplatin against liver carcinoma HepG2 (Table 4) [76]. Complex **27** shows intermediate activity that surpasses **25a** but does not reach the efficacy of complex **26** or cisplatin [76]. Overall, the comparison shows a clear progression in potency, with **25a** as a weaker performer, **27** displaying moderate activity, and complex **26** emerging as the promising analogue relative to cisplatin.

The SAR of amino acid-based gold(I) complexes indicates that biological activity is primarily governed by gold–ligand coordination rather than the amino acid scaffold itself. Au-S coordinated complexes consistently show higher cytotoxicity than Au–X (Cl, Br) analogues, highlighting the importance of strong sulphur–gold interactions for intracellular activity. In contrast, dipeptide-based complexes exhibit reduced potency, suggesting that increased peptide bulk limits cellular uptake. Overall, optimal activity in this class arises from strong Au–S binding combined with minimal peptide character. Similarly, incorporation of amino acid in NHC gold(I) complexes demonstrates how biocompatible modifications can modulate cytotoxicity, potentially through enhanced interactions with thiol-containing biomolecules.”

## 6. Natural Product-Based Heterobimetallic Gold(I) Complexes

In recent years, heterobimetallic complexes incorporating gold(I) centres have emerged as an effective approach to enhance anticancer activity by combining two distinct metal centres within a single molecular scaffold, often leveraging metal–metal synergistic mechanisms. These complexes benefit from enhanced chemical stability, favourable photophysical properties, improved bioavailability, and diverse pharmacological effects imparted by the heteronuclear arrangement [77,78].

Moreover, the addition of a second metal such as ruthenium, iron, or titanium, alongside gold(I), has been shown to improve cellular uptake and selectivity, often leading to superior anticancer efficacy compared to monometallic analogues [79]. When conjugated with biologically active natural products, these systems not only exploit the inherent cytotoxic and anti-inflammatory properties of gold(I), but also engage distinct mechanisms of action, including TrxR inhibition, ROS generation, and Endoplasmic Reticulum Stress. This section highlights the growing relevance of natural product-derived heterometallic gold(I) complexes, discussing key examples and their implications for next-generation cancer therapy.

**Figure 6:**
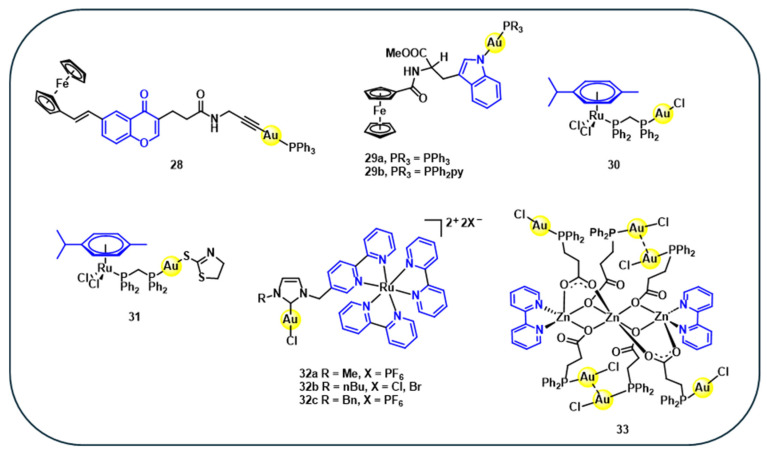
Structures of natural product-based heterometallic gold(I) complexes **28–33**.

To evaluate whether incorporating a second metal enhances biological activity compared to monometallic analogues, a gold(I)–iron heterobimetallic complex **28** (Figure 6), consisting of gold(I) and iron centres coordinated to a natural product-derived chromone ligand, has been synthesised. Despite its innovative heteronuclear structure, complex **28** exhibits lower cytotoxicity relative to its monometallic gold counterpart. However, its cytotoxicity against MCF-7 (IC_50_ = 11.0 µM) and CCRF-CEM (IC_50_ = 3.8 µM) cancer cell lines was better than that of auranofin, which showed IC_50_ values of 13.1 µM and 6.0 µM, respectively (Table 5) [80]. The experimental findings indicated that having multiple metals within a single molecular framework did not necessarily enhance the bioactivity of the Au-Fe bimetallic complex **28** [81,82]. To expand the scope of heterobimetallic gold(I) complexes, **29a–b** (Figure 6) are synthesised to investigate their synergistic effects. In these complexes, gold-phosphine units are linked through an Au-N coordination to the indole moiety of the tryptophan ester ligands bearing ferrocene [83]. The cytotoxicity studies in cervical carcinoma (HeLa) cells showed that the iron monoconjugate exhibits negligible cytotoxicity (IC_50_ > 1000 µM), whereas corresponding gold–ferrocene heterobimetallic complexes **29a–b** exhibit improved antiproliferative activity in HeLa cells with IC_50_ values of 32 µM and 22 µM, respectively (Table 5) [83]. These results reveales that the ferrocene bioconjugates alone have minimal antiproliferative effects. In contrast, the heterometallic gold(I) complexes exhibit pronounced cytotoxicity and limit the proliferation of multiple cancer cell lines in vitro, highlighting the cooperative effect between the gold and ferrocene moieties in enhancing anticancer activity [83].

In a similar approach, gold(I)–ruthenium(II) (Au–Ru) heterobimetallic complexes **30** and **31** (Figure 6) incorporating the natural product *p*-cymene are synthesised and evaluated for anticancer activities. These complexes exhibit significantly higher antiproliferative activity (IC_50_ = 4.6–6.5 μM) than their monometallic and dimer ruthenium analogues (IC_50_ = 21.9–73.1 μM) (Table 5). The increased cytotoxicity is mainly attributed to the presence of the gold(I) centre, likely due to its strong affinity for thiol and selenol-containing proteins. Moreover, these complexes display improved cancer cell selectivity and potential protein interactions, suggesting mechanisms beyond traditional nucleic acid targeting [84].

**Figure 7:**
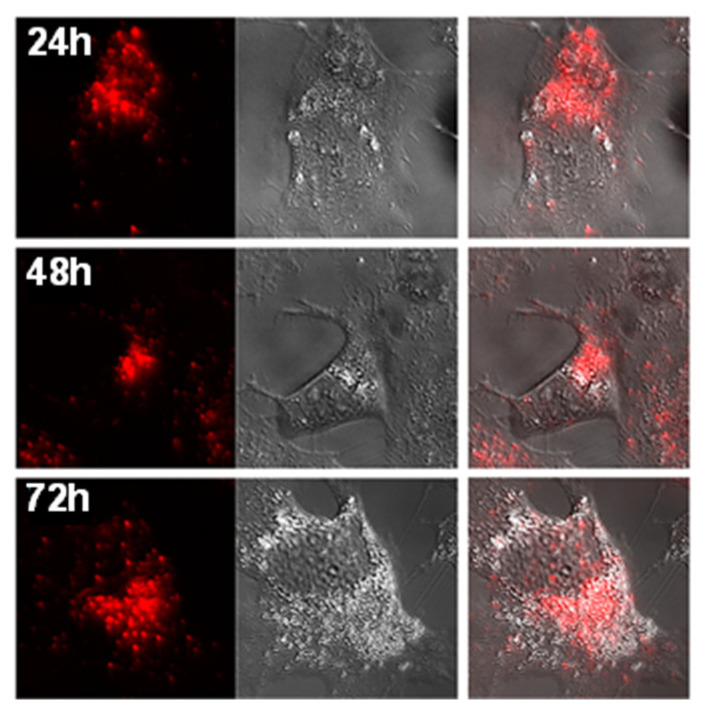
Time-course confocal imaging of Hep3B cells treated with **32b** with 10 µM. Fluorescence collected at excitation at 458 nm and emission at 516–670 nm. Images obtained at 24, 48, and 72 h show rapid uptake with strong cytoplasmic/perinuclear localisation. Fluorescence from **32b** is shown in red, alongside transmission (bright-field) images (middle panels) and merged images (right panels) [85] (reproduced with permission).

In a related study, three heterobimetallic gold(I)–ruthenium(II) complexes **32a–c** (Figure 6) featuring heteroditopic bipyridine–NHC ligands are synthesised by combining a gold–NHC unit and a Ru(bipy)_3_ building block. Since the gold–NHC complex is less stable than the Ru^II^(bipy)_3_ complex and is prone to decomposition, which leads to gold nanoparticles, the synthesis is carried out by first coordinating ruthenium with the substituted imidazolium salts, followed by the transformation of the imidazolium into the NHC–gold(I) unit via the silver oxide route [85]. Biological evaluation against human hepatocellular carcinoma (Hep3B) cells shows that the gold-containing complexes exhibit high cytotoxicity (IC_50_ =17– 31 µM), compared to the ruthenium–imidazolium precursor, which displayed low cytotoxicity (IC_50_ > 100 µM) (Table 5), highlighting the role of the gold for cytotoxic activity. The most active complex **32b** displayed favourable photophysical properties and was used in fluorescence microscopy experiments, revealing rapid cellular uptake and cytoplasmic localisation in Hep3B cells (Figure 7). Also, the emission-spectrum findings suggest that the gold–ruthenium complexes gradually release gold cations into the cells, resulting in the formation of corresponding ruthenium complexes and ultimately leading to cell death. This supports the observation that the presence of gold is crucial for the cytotoxic activity [85]. Confocal fluorescence imaging confirmed that **32b** complex yielded a bright, predominantly cytoplasmic/perinuclear signal that persisted over 24–72 h (Figure 7), supporting the theory that intracellular Au^+^ release and conversion to the Ru-only species linked to cytotoxicity [85]. These findings support the potential of heterometallic gold(I) complexes as dual theranostic agents.

**Figure 8:**
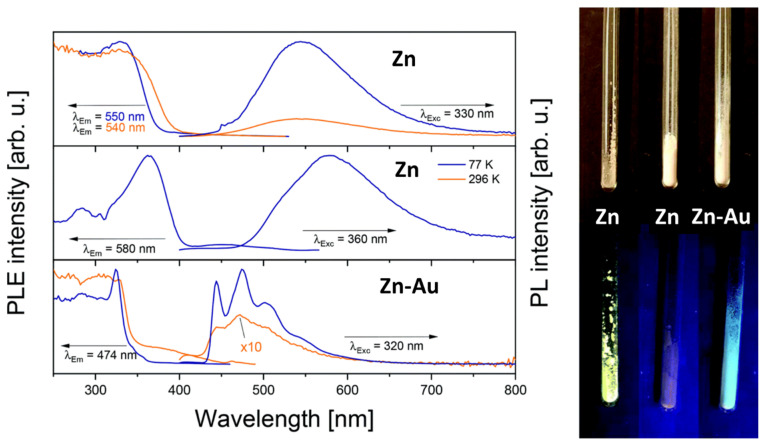
Solid-state photoluminescence (PL) and photoluminescence excitation (PLE) spectra of complex **33** [86] (reproduced with permission).

In a recent study, a hexanuclear zinc–gold [Zn_3_Au_6_] heterobimetallic complex **33** (Figure 6) has been synthesised, featuring carboxyl-functionalized phosphine ligands derived from natural product-like structures. These complexes exhibit strong photoluminescence at both room temperature and 77 K, demonstrating their potential as luminescent probes for biomedical imaging [86]. The luminescence behaviour differs markedly from that of the zinc metallo ligand alone, with the Zn-Au heterobimetallic showing distinct emission spectra at both room temperature and 77 K, indicating aurophilic interactions (Figure 8).

These findings underscore that incorporating two distinct metal centres into natural product-based frameworks can significantly enhance biological activity. However, these synergistic effects are not universally guaranteed; the development of heterometallic systems presents a promising strategy for fine-tuning the therapeutic properties of monometallic compounds. These studies exemplify a growing trend toward multifunctional gold(I)-based systems, in which therapeutic activity, intracellular tracking, and mechanistic insight are integrated into a single molecular framework.

Across all classes, gold(I) coordination with bioactive ligands consistently outperforms the corresponding parent ligands. In most cases, free ligands act as weakly active or biologically inert scaffolds, whereas gold(I) complexes display enhanced potency, expanded mechanistic profiles, and improved activity in resistant cancer models. These comparisons highlight the role of gold(I)as a central contributor to therapeutic efficacy.

## 7. Conclusions

This review highlights the importance of diverse nature-inspired scaffolds, including heterobimetallic systems, in the development of gold(I)-based anticancer agents. Each scaffold class contributes distinct structural and functional attributes that shape biological activity and therapeutic applications. Nitrogen-containing heterocycles remain central due to their synthetic flexibility and strong target interactions, while stilbenes scaffolds provide valuable antimitotic and anti-vascular effects. Amino acid-based systems offer improved biocompatibility, whereas heterobimetallic complexes introduce synergistic mechanisms that expand pharmacological possibilities.

Across all scaffold ligands, ligand design emerges as a central determinant of complex stability, target engagement, and biological outcome, acting in coordination with the gold(I) centre. Structure–activity trends indicate that the combined properties of the gold(I) centre and ligand collectively govern anticancer efficacy and increasingly determine the biological mechanism of action, including multifunctional effects such as imaging-enabled cellular tracking. Despite these advances, challenges related to synthetic scalability, pharmacokinetics, systemic toxicity, long-term stability, and translational reliability continue to impede clinical progression. Future research should prioritise mechanistically informed ligand design, integration of computational and structure-based approaches, exploration of targets beyond TrxR, and incorporation of targeted delivery strategies. In vivo validation and comparative studies against clinically relevant agents will be essential to support translation. Notably, only a limited number of systems, such as selected pyridine-based complexes and a combretastatin-derived stilbene complex, have advanced to in vivo studies, where promising antitumour and antivascular effects with acceptable tolerability have been reported. These early findings highlight both the translational promise of nature-inspired gold(I) complexes and the need for more comprehensive pharmacokinetic, biodistribution, and long-term toxicity studies to support clinical advancement.

## Figures and Tables

**Table 1:** In vitro anticancer activity (IC_50_ µM) of nitrogen-containing gold(I) complexes **1–6**.

Complex	5637	T24	CH1	SK-OV-3	SW480	Hela	Ref.
**1**	0.17	2.59	-	-	-	-	[47]
**2**	2.56	12.4	-	-	-	-	[47]
**6**	-	-	1.5	10	9.3	4.6	[48]
Cisplatin	-	-	0.16	1.9	3.5	0.37	[48]

Cancer cell lines: 5637 and T24 (bladder cancer), CH1 and SKOV-3 (ovarian cancer), SW480 (colon cancer), and HeLa (cervical cancer).

**Table 2:** In vitro anticancer activity (IC_50_ µM) of heterocyclic-based gold(I) complexes **7–17**.

Complex	GXF 251	LXFA 629	MAXF 401	22RV1	UXF 1138	OVXF 899	RXF 486	Ref.
**7**	2.28	7.371	2.246	ND	3.377	20.212	3.046	[63]
**9**	0.219	0.131	0.075	0.239	0.026	2.483	4.13	[63]
**10**	0.006	0.005	0.003	0.09	0.006	0.564	0.871	[63]
**11**	0.056	0.027	0.008	0.022	0.011	1.472	1.062	[63]
**12**	0.005	0.003	0.003	0.004	0.004	0.396	0.595	[63]
**13**	0.029	0.017	0.009	0.023	0.017	1.472	1.062	[63]
**14**	0.058	0.019	0.008	0.022	0.019	1.066	2.389	[63]
**15**	0.005	0.006	0.003	0.01	0.007	0.529	1.031	[63]
**16**	37.05	45.2	11.52	ND	16.78	44.95	18.18	[63]
**17**	5.47	3.94	5.27	12.72	1.25	5.43	3.86	[63]
Parent **7–17**	100	100	100	100	100	100	100	[63]
Cisplatin	4.97	4.71	0.68	3.57	2.77	2.54	8.79	[63]

Cancer cell line: GXF 251 (gastric cancer), LXFA 629 (lung cancer), MAXF 401 (mammary cancer), 22RV1 (prostate cancer), UXF 1138 (cancer of the uterus), OVXF 899 (ovarian cancer), and RXF 486 (renal cancer). ND = not determinable.

**Table 3:** In vitro anticancer activity (IC_50_ µM 72 h) of stilbene-derived gold(I) complexes **18–24**.

	518A2	Panc-1	HCT-116	HT-29	MCF-7	MCF-7/Topo	MCF-7/Topo + FTC	KB-V1/Vbl	KB-V1/Vbl + Ver	HL-60	HF	MDA-MB 231	Ref.
**18a**	0.46	0.40	0.30	0.08		0.18	0.19	7.7	2.9	-		-	[71]
**18b**	0.31	0.16	0.11	0.15		0.06	0.09	2.0	0.23	-		-	[71]
**18c**	0.37	0.26	0.11	0.13		0.15	0.12	3.9	0.45	-		-	[71]
**18d**	0.43	0.19	0.18	0.06		0.08	0.13	3.7	1.3	-		-	[71]
**18e**	0.43	0.36	0.07	0.10		0.06	0.08	1.1	0.22	-		-	[71]
**18f**	0.23	0.18	0.12	0.16		0.10	0.10	1.5	0.20	-		-	[71]
Parent **18**	0.02	ND	ND	3.6		0.50	ND	<0.01	ND	-		-	[71]
**19a**	12	-	-	20	-	-	-	37	-	8.2	>100	-	[72]
**19b**	17	-	-	11	-	-	-	39	-	6.9	>50	-	[72]
Parent **19a–b**	>100	-	-	>100	-	-	-	>100	-	>50	>100	-	[72]
**19c**	20	-	-	14	-	-	-	>50	-	10	37	-	[72]
**19d**	>50	-	-	23	-	-	-	>50	-	23	31	-	[72]
**20a**	-	-	-	0.43	0.17	-	-	-	-	-	-	0.54	[73]
**20b**	-	-	-	0.23	0.10	-	-	-	-	-	-	0.34	[73]
**20c**	-	-	-	0.47	0.30	-	-	-	-	-	-	1.55	[73]
**21a**	-	-	-	0.42	0.22	-	-	-	-	-	-	0.70	[73]
**21b**	-	-	-	0.37	0.21	-	-	-	-	-	-	0.67	[73]
**22a**	-	-	-	3.19	1.26	-	-	-	-	-	-	1.44	[73]
**22b**	-	-	-	3.22	0.81	-	-	-	-	-	-	1.34	[73]
**23a**	-	-	-	0.36	0.13	-	-	-	-	-	-	0.56	[73]
**23b**	-	-	-	0.62	0.15	-	-	-	-	-	-	0.40	[73]
**24a**	-	-	-	3.1	1.2	-	-	-	-	-	-	2.4	[74]
**24b**	-	-	-	4.2	1.6	-	-	-	-	-	-	2.9	[74]
**24c**	-	-	-	2.9	1.4	-	-	-	-	-	-	3.7	[74]
**24d**	-	-	-	3.3	0.8	-	-	-	-	-	-	1.7	[74]
**24e**	-	-	-	4.2	3.1	-	-	-	-	-	-	6.4	[74]
**24f**	-	-	-	2.3	1.1	-	-	-	-	-	-	3.9	[74]
**24g**	-	-	-	3.3	0.87	-	-	-	-	-	-	3.1	[74]
**24h**	-	-	-	17.0	4.5	-	-	-	-	-	-	>20	[74]
**24i**	-	-	-	4.3	2.6	-	-	-	-	-	-	3.3	[74]
**24j**	-	-	-	3.2	1.8	-	-	-	-	-	-	2.4	[74]
Auranofin	-	-	-	2.6	1.1	-	-	-	-	-	-	ND	[74]
Cisplatin	-	-	-	4.1	1.6	-	-	-	-	-	-	ND	[74]
5-FU	-	-	-	7.3	4.7	-	-	-	-	-	-	-	[74]

Cancerous cell line: 518A2 (melanoma), Panc-1 (pancreatic ductular adenocarcinoma), HCT-116 and HT-29 (colon carcinomas), MCF and MDA-MB 231 (breast cancer), MCF-7/Topo breast adenocarcinoma (optionally pretreated with 1.2 μM fumitremorgin C for 24 h), KB-V1/Vbl (cervix carcinoma) (optionally pretreated with 24 μM verapamil HCl for 24 h), HL-60 (human leukaemia) Noncancerous cell line: HF (non-malignant foreskin fibroblasts). ND: not determined.

**Table 4:** In vitro anticancer activity (IC_50_ µM 72 h) of amino acid-based complexes **25–27**.

Complex	Hela	HepG2	HT-29	Ref.
**25a**	45.5	61.4	63.8	[76]
**26**	3.4	15.2	10.5	[76]
**27**	17.3	28.1	26.8	[76]
Cisplatin	3.5	1.1	7.9	[76]

Cancer cell lines: HeLa (human cervical epitheloid carcinoma), HT-29 (human colon adenocarcinoma, grade II), and HepG2 (human hepatocellular liver carcinoma).

**Table 5:** In vitro anticancer activity (IC_50,_ µM; 24–48 h) of heterometallic gold(I) complexes.

Complex	HepG2	MCF-7	MDA-MB-231	CCRF-CEM	HeLa	N1E-115	HCT116	Hep3B	L929	Ref.
**28** (Au-Fe)	>120	11.0	49.7	3.8	-	-	-	-	-	[80]
Auranofin	50.0	13.1	3.0	6.0	-	-	-	-	-	[80]
**29a** (Au-Fe)	-	15	-	-	32	27	-	-	-	[83]
**29b** (Au-Fe)	-	45	-	-	22	26	-	-	-	[83]
Parent **29** (Fe)	-	NT	-	-	>1000	NT	-	-	-	[83]
**30** (Au-Ru)	-	-	-	-	-	-	4.6	-	36.1	[84]
**31** (Au-Ru)	-	-	-	-	-	-	6.5	-	48.6	[84]
Parent **30–31** (Ru-Ru)	-	-	-	-	-	-	73.7	-	243.4	[84]
Parent **30–31** (Ru)	-	-	-	-	-	-	21.9	-	39.6	[84]
**32a** (Ru-Au)	-	-	-	-	-	-	-	-	-	[85]
**32b** (Ru-Au)	-	-	-	-	-	-	-	-	-	[85]
**32c** (Ru-Au)	-	-	-	-	-	-	-	18.9	-	[85]
Parent **32** (Ru)	-	-	-	-	-	-	-	>100	-	[85]
sorafenib		-			-	-	-	7.2	-	[85]

Cancer cell lines: HepG2 (hepatocellular carcinoma), MCF-7 (estrogen-responsive breast cancer), MDA-MB-231 (estrogen-unresponsive breast cancer), CCRF-CEM (T-cell lymphoblastic leukaemia), HeLa (cervical cancer), NIE-115 (murine neuroblastoma, derived from C-1300 mouse sympathetic ganglion neurons), and HCT116 (colon carcinoma)**,** Hep3b (human hepatocellular carcinoma) Non-cancerous cell line: L929 (non-tumorigenic mouse fibroblast). NT– non tested.

## Data Availability

Not applicable.

## Abbreviations

NHC	N-heterocyclic carbene
TrxR	Thioredoxin reductase
5637	Human bladder cancer cell lines
MDA-MB-231 and MCF-7	Breast cancer cells
Hep3B	Human hepatocellular carcinoma
5-FU	5-fluorouracil
ROS	Reactive oxygen species
SAR	Structure–activity relationships
